# Time-dependent spatial specificity of high-resolution fMRI: insights into mesoscopic neurovascular coupling

**DOI:** 10.1098/rstb.2019.0623

**Published:** 2020-11-16

**Authors:** Mitsuhiro Fukuda, Alexander J. Poplawsky, Seong-Gi Kim

**Affiliations:** 1 Department of Radiology, University of Pittsburgh, Pittsburgh, PA, USA; 2 Center for Neuroscience Imaging Research, Institute for Basic Science, Suwon, South Korea; 3 Department of Biomedical Engineering, Sungkyunkwan University, Suwon, South Korea

**Keywords:** blood oxygenation level-dependent, cerebral blood volume, cerebral blood flow, column, layer

## Abstract

High-resolution functional magnetic resonance imaging (fMRI) is becoming increasingly popular because of the growing availability of ultra-high magnetic fields which are capable of improving sensitivity and spatial resolution. However, it is debatable whether increased spatial resolutions for haemodynamic-based techniques, like fMRI, can accurately detect the true location of neuronal activity. We have addressed this issue in functional columns and layers of animals with haemoglobin-based optical imaging and different fMRI contrasts, such as blood oxygenation level-dependent, cerebral blood flow and cerebral blood volume fMRI. In this review, we describe empirical evidence primarily from our own studies on how well these fMRI signals are spatially specific to the neuronally active site and discuss insights into neurovascular coupling at the mesoscale.

This article is part of the theme issue ‘Key relationships between non-invasive functional neuroimaging and the underlying neuronal activity’.

## 1. Introduction

Increased neuronal activity in the brain is accompanied by changes in oxygen metabolism, blood flow, and blood volume, which can be captured with functional magnetic resonance imaging (fMRI) as blood oxygenation level-dependent (BOLD), cerebral blood flow (CBF) or cerebral blood volume (CBV) changes. Owing to its noninvasiveness and flexibility, fMRI occupies a major position to probe human brain function. However, the interpretability of fMRI maps is not straightforward because it indirectly infers neuronal activity from vascular responses. When increased neuronal activity occurs in the vicinity of microvessels, upstream arteries dilate to increase local blood oxygenation, which subsequently drains through downstream veins. Therefore, a location mismatch between the site of increased neuronal activity and the site of vascular responses measured by fMRI varies depending on how far these sites are separated and which vascular compartment the fMRI technique is sensitive to. For example, BOLD fMRI is biased toward venous blood, while CBV is weighted toward arterial blood (see [1] for a detailed review of fMRI biophysical properties). In particular, the mismatch between active neuronal and vascular sites is problematic at high resolutions (less than 1 mm isotropic in humans, approximately 0.1-mm in-plane resolution in animals) because different vascular compartments do not reside in the same voxel, unlike for low-resolution fMRI. Therefore, one of the outstanding issues of high-resolution fMRI is how accurately the signal changes mark the true site of increased neuronal activity. To examine this spatial specificity of fMRI, activation must be measured within well-defined neuronal circuits, like functional columns or layers, and validated with other well-established complementary techniques, including optical imaging and electrophysiology, in animal models. In this review, we will focus on findings based primarily on our studies of iso-orientation columns and cortical layers in the cat visual cortex and provide insights into neurovascular coupling at the mesoscopic scale. Topics discussed here are relevant to human fMRI application.

## 2. How spatially specific is the vascular architecture?

Since fMRI measures a vascular response, the vascular architecture imposes constraints on the spatial resolution of fMRI. It is, therefore, important to briefly understand how the vasculature is organized in cortical layers and columns (see [2] for a detailed review). The cortical vasculature can be divided into two main compartments: pial and intracortical networks. The pial vessels consist of large arteries and veins that run above the cortical surface and contribute mostly non-specific vascular responses. The intracortical vascular network can be divided into the penetrating arteries and emerging veins that travel perpendicular to the cortical layers and then branch out into microvessels, including arterioles, capillaries and venules. Oxygen is delivered by all microvessels, including arterioles and venules [3]. Since the oxygen saturation level of small pial arteries is 82% [4], this level will be lower in smaller intracortical arterioles and, therefore, these vessels could contribute to the BOLD fMRI response depending on the degree of oxygenation change. The median diameter for penetrating vessels is 11 µm for arteries and 9 µm for veins in the mouse barrel cortex [5]. The median distance between penetrating arteries is approximately 120 µm for arteries and approximately 100 µm for veins. In addition, the number of branching points peaks at cortical layer IV for penetrating arteries, but peaks near the surface for emerging veins [5]. Interestingly, emerging veins are more numerous than penetrating arteries in rodents [5,6], but have the opposite trend in primates [7]. In addition, the density of penetrating arteries increases with phylogenetically higher-order animals [8]. A single artery in the rodent barrel cortex drains to several veins while a single vein receives from several arteries [9]; thus, measuring from veins is more spatially non-specific. The vascular territory of penetrating arteries is similar to the location of the neuronal barrels in layer IV (less than 500 µm). However, a spatial association between the intracortical vasculature and functional structures is debatable, with some studies finding such a correlation [10–12], while others did not [5,13]. Microvessels are often considered as vessels with a diameter of less than 6 µm, regardless of the species. The mean distance from the centre of neuronal cell bodies to the nearest microvessel is 14.6 µm [6]. However, the microvascular density does not correlate well with the layer-to-layer variations in neuronal density [5,6]. Instead it appears to be specialized to the cortical layer oxidative [7,11] and glucose [14–16] metabolism, with the highest density often found in the thalamocortical input layer IV for primary sensory cortices [7,11,12,15].

## 3. Spatial and temporal properties of vascular responses revealed by optical intrinsic signal imaging

**Figure 1.**
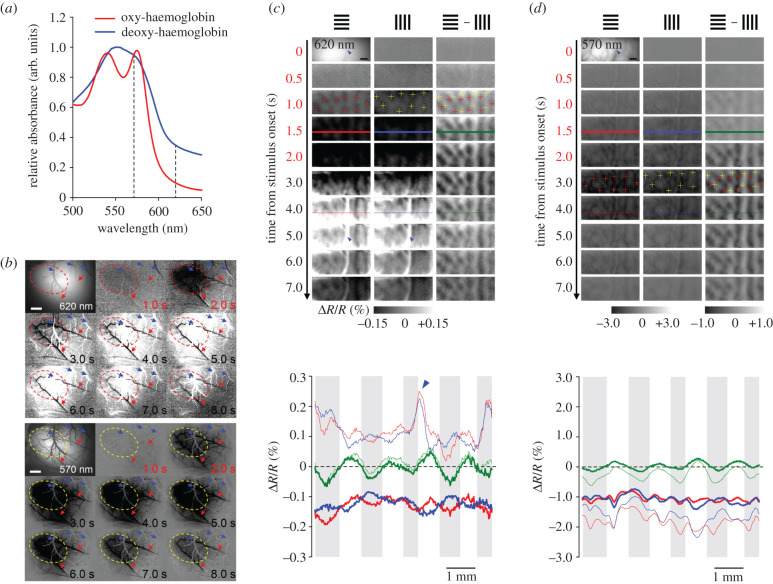
Optical intrinsic signal imaging. (*a*) Absorption spectra of oxy- (red) and deoxy-haemoglobin (deoxyHb) (blue). OISI at 570 nm (haemoglobin isosbestic point) and 620 nm (indicated by dotted lines) are weighted toward totalHb and deoxyHb, respectively. Spectral decomposition is often used for better separation of each component. However, small changes are controversial, such as with the initial dip. From [17]. (*b*) Time-dependent functional maps of deoxyHb- (top) and totalHb-weighted OISI (bottom) concurrently measured at 620 and 570 nm, respectively, from the visual cortex of an isoflurane-anaesthetized rat. Stimulation: 2 s full-field, square-wave grating; bottom right of each frame: time from stimulation onset (time stamps in red: stimulation period); top left: baseline image with pial vessels; dashed contours: neuronally active regions; red arrows: pial arteries; blue arrows: pial veins; scale bar: 1 mm. From M. Fukuda 2010, unpublished data. (*c,d*) Time-dependent functional maps (top) and cortical profiles (bottom) of deoxyHb- (*c*) and totalHb-weighted OISI (*d*) from the cat visual cortex. Stimulation: 2 s full-field, square-wave gratings; time stamps in red: stimulation period. *Top panel*, spatio-temporal development of OIS maps responding to horizontal (left) and vertical gratings (middle), and their differential (horizontal − vertical) maps (right column). Top left: pial vessel images with a focal plane of 700 µm below the surface; blue arrowheads: large pial veins; scale bar: 1 mm; *y*-axis: time from stimulation onset; ‘+’ markers: iso-orientation domains determined on the initial dip of deoxyHb-weighted OIS maps, then overlaid on totalHb-weighted OIS maps as a place reference, red and yellow ‘+’: iso-orientation domains for horizontal and vertical gratings, respectively. *Bottom panel,* line profiles from 1.5 s (thick lines) and 4.0 s OIS maps (thin lines). Red and blue traces: OIS evoked by horizontal and vertical gratings, respectively; green traces: differential profile (red − blue); horizontal grey bars: horizontal iso-orientation domains determined by negative deflections of green traces at 1.5 s in deoxyHb-weighted OISI. Adapted from [17].

Optical intrinsic signal imaging (OISI) is often used as a surrogate of fMRI for studying spatio-temporal haemodynamics since both measure the oxygenation state of haemoglobin. OISI measures visible light reflectance changes of haemoglobin (Δ*R*/*R*) and is capable of higher spatial and temporal resolutions, but is limited to the brain surface. OISI at 620 nm wavelength is weighted toward deoxy-haemoglobin (deoxyHb) changes, like BOLD fMRI, while OISI at 570 nm is weighted toward total haemoglobin (totalHb) and is related to CBV-weighted fMRI (figure 1*a*). For example, let us examine the typical spatio-temporal dynamics of OISI responses evoked by a 2 s visual stimulation in the rat visual cortex (figure 1*b*). While deoxyHb-weighted OISI consists of an initial darkening of the cortex (i.e. increased deoxyHb; herein referred to as the ‘initial dip’) followed by lightening (i.e. decreased deoxyHb) (top panel), the totalHb-weighted OISI only darkens (i.e. increased blood volume) (bottom panel). The initial dip is localized well to the neuronally active region (see red dashed contour at 1 s), and is presumably caused by increased oxygen consumption of activated neurons rather than increased blood volume since no changes are observed at 1 s with totalHb-weighted OISI. At 2 s, pial arteries (red arrows) begin to dilate (darkening in both OISI modalities). At 3 s, deoxyHb begins to decrease in pial veins (blue arrows) and tissue, and spreads almost over the whole imaging area by 4–6 s, similar to the increases in blood volume during this time. Thus, the decreases in deoxyHb (i.e. positive BOLD) are presumably caused by increases in oxygenated blood flow (i.e. hyperoxygenation) in the draining veins. Note that no pial veins dilate (blue arrows in 570 nm images), unlike pial arteries (red arrows in 570 nm images), consistent with CBV responses measured with fMRI [18]; but venous vessel dilation can occur with a long stimulus duration [19,20] or under medetomidine anaesthesia [21]. Interestingly, blood volume changes appear to be more confined to the neuronally active region long after the stimulation offset (e.g. 3 s versus 7 s). Although this OISI example provides several important insights on vascular responses, it is not suitable for understanding highly spatially specific responses.

**Figure 2.**
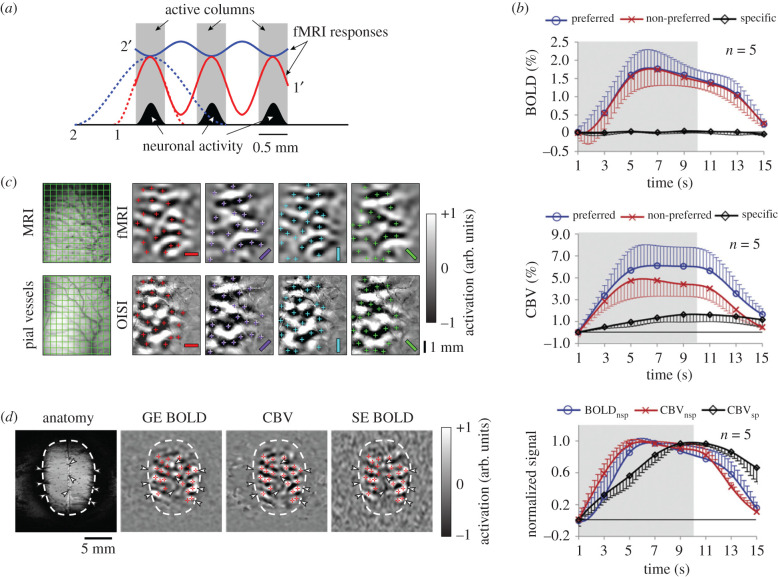
Functional column mapping of iso-orientation domains with fMRI. (*a*) Hypothetical spatial profiles of haemodynamic responses during single-column activation (dotted lines) with narrow (1, red) and broad (2, blue) point spread functions (PSFs) and during multiple-column activations (solid lines 1′ and 2′) with the narrow (1, red) and broad (2, blue) PSFs. Visual stimulation elicits neuronal activity only (black bell-shaped curves) within active columns (grey bars), but not in inactive columns (white space). When a single column is active (dotted lines 1 and 2), the highest response is located within the active column regardless of the PSF. But if multiple columns are active simultaneously (solid lines 1′ and 2′), the location of the response peaks is dependent on the PSF. For example, the response peaks either in active columns for a narrow PSF (profile 1′) or in inactive columns for a broader PSF (profile 2′). Adapted from [24]. (*b*) Time courses of orientation-specific and -non-specific BOLD (top) and CE-CBV fMRI signals (middle) responding to 10-s grating stimulation in the cat visual cortex. The orientation-specific response (black) was determined by the difference between two responses (preferred orientation response (blue) − non-preferred (red)). *Bottom panel*, temporal dynamics of the normalized non-specific and specific responses. Note that the orientation-specific CBV response (CBV_sp_, black) is slower than the non-specific CBV (CBV_nsp_, red) and BOLD (BOLD_nsp_, blue) responses and is sustained after the stimulation. Error bars: s.d. (*n* = 5 cats); grey bars: stimulation period. Adapted from [25]. (*c*) Comparison of iso-orientation maps between CE-CBV fMRI (top row) and totalHb-weighted OISI (bottom row) from the same cat. Stimulation: continuous cyclic stimulation of rotating gratings and Fourier analysis to remove common activations. *First column*, pial vessel patterns obtained with anatomical MRI (top) and optical imaging (bottom), used for image co-registration. Grids are 0.5×0.5 mm^2^. *Second–last columns*, iso-orientation maps at stimulus bar orientations of 0 (horizontal bars), 45, 90 and 135°. ‘+’ markers: iso-orientation domains determined on totalHb-weighted OIS maps, then overlaid on fMRI maps as a place reference. Adapted from [24]. (*d*) Comparisons of GE-BOLD, CBV and SE-BOLD fMRI iso-orientation maps obtained from the same cat during cyclic stimulation of rotating gratings. Red ‘+’ markers indicate iso-orientation domains determined from CBV maps, then overlaid on GE- and SE-BOLD maps as a place reference. White arrowheads: large veins. Adapted from [26].

To determine submillimetre-scale neuronal specificity of haemodynamic responses, we next examined OISI for iso-orientation columns in the phylogenetically higher-ordered cat primary visual cortex [22,23]. Neurons that respond to the same orientation of a full-field square-wave grating stimulus (i.e. bars) are clustered in cortical functional columns. The size of a column is approximately 500 µm, with an inter-columnar distance of approximately 1 mm. The spatial specificity of haemodynamic responses was determined by investigating the capability of such techniques to resolve individual iso-orientation columns when multiple columns were simultaneously activated (figure 2*a*). For deoxyHb-weighted OISI (figure 1*c*, top panel), visual stimulation initially induced increases, then decreases in deoxyHb regardless of whether the stimulus gratings were horizontal (left column) or vertical (middle). In these single-stimulus conditions, iso-orientation domains (‘+’ on images at 1.0 s) were only visible during the initial dip. Electrophysiological studies confirmed that neurons located here fire action potentials only to a preferred orientation [27]. However, iso-orientation domains were present after 1.0 s in differential images (right column) only when common responses, including from pial veins (blue arrowhead), were removed, as seen in their profiles (bottom panel). Interestingly, the differential profiles at 1.5 and 4.0 s (green lines) both showed increased deoxyHb in the active column, suggesting that the hyperoxygenation was greater in the inactive relative to the active column (e.g. compare profiles at 4 s).

To examine the spatial specificity of CBV, totalHb-weighted OISI (figure 1*d*, top panel) were acquired. Iso-orientation domains (e.g. ‘+’ on images at 3.0 s) were not visible in their single-condition images (left and middle columns), but became apparent after subtraction (right column). The response profiles (bottom panel) were also alike, particularly at the earlier time point (thicker red and blue traces), with the small orientation-specific modulations (green traces) being largely masked by the common responses. Importantly, the iso-orientation domains from the totalHb-weighted OIS differential maps were observed for early and late time points and matched the deoxyHb initial dip maps, suggesting that blood volume changes are regulated at a submillimetre scale. In summary, iso-orientation columnar maps obtained by OISI agree well with the electrophysiologically determined orientation selectivity [27], thus it can be used as the gold standard of neuronally active columns for comparison with columnar-resolution fMRI.

## 4. Spatial specificity of fMRI examined with the functional column model

Spatial specificity of fMRI seems high regardless of the haemodynamic point spread function (PSF) when only a single column (e.g. single whisker barrel or digit somatosensory area in rodents) is activated (figure 2*a*, dotted lines, 1 and 2), but is dependent on the PSF when multiple functional sites are adjacently activated, such as in orientation columns (figure 2*a*, solid lines, 1′ and 2′). With inferences from OISI studies, iso-orientation columns were first mapped with gradient echo (GE)-BOLD fMRI using the initial dip [28] and perfusion-based arterial spin labelling (ASL) fMRI, which measures CBF changes [29]. The improved spatial specificity of ASL fMRI is due to its sensitivity to arterial spin labelled water that perfuses into the tissue, prominently through microvessels. However, the positive BOLD response evoked by 10 s long stimulations could not resolve the iso-orientation columns even with the differential method (figure 2*b*, top panel) [25,28]; responses to the preferred orientation in a given column (blue trace) had the same amplitude as responses to the non-preferred orientation (red), resulting in no differential orientation-specific signal (black). In contrast to BOLD fMRI, contrast-enhanced plasma blood volume-weighted (CE-CBV) fMRI which used an exogenous intravascular contrast agent did detect the iso-orientation columns (figure 2*b,* middle panel). The CBV response to the preferred orientation in a given column (blue trace) was larger than the response to the non-preferred orientation (red), resulting in orientation-specific signal (black). It was evident that employing a longer stimulus should improve the spatial specificity since relative non-specific responses decreased with the stimulus duration, while the specific responses increased or remained constant (figure 2*b*, bottom panel). This dynamic property is somewhat similar to totalHb-based OISI (figure 1*d,* bottom panel, 1.5 versus 4.0 s).

Orientation-specific CE-CBV fMRI maps may or may not be accurately reporting active columns, depending on the intrinsic PSF (figure 2*a*). To resolve this issue, CE-CBV fMRI and neural-specific OISI iso-orientation maps were obtained in the same animals with continuous cyclic stimulation and Fourier analysis [24]. Iso-orientation maps from both imaging methods were remarkably similar after image registration (figure 2*c*), demonstrating that increased plasma blood volume (measured by CE-CBV fMRI) and totalHb content (measured by 570 nm OISI) occurred in the same iso-orientation domains, and that orientation-specific blood volume signals should stem mainly from dilation of intracortical microvessels.

Next, we tested whether the continuous cyclic stimulation could resolve the iso-orientation columns using the positive BOLD fMRI response [26]. Both GE- and spin echo (SE)-BOLD maps were essentially the same as the CBV maps (figure 2*d*), meaning that the hyperoxygenation phase of the BOLD response was specific to the neuronally active columns (i.e. profile 1′ in figure 2*a*). This is contradictory to our deoxyHb-weighted OISI results (see §3 and figure 1*c*); therefore, either the PSF of the positive BOLD signal is narrower than that of deoxyHb-weighted OISI during the hyperoxygenation period or the latter is contaminated by orientation-specific totalHb signal. Note that SE-BOLD fMRI is less sensitive to changes in larger pial vessels (white arrowheads in figure 2*d*) and, therefore, appears more specific than GE-BOLD fMRI.

The detectability of functional columnar maps is dependent on spatial specificity and sensitivity (i.e. contrast-to-noise ratio). Spatial specificity can be described as a ratio between 0 (no specificity) and 1 (complete specificity). The spatial specificity of SE-BOLD fMRI is slightly higher than that of GE-BOLD (0.26 versus 0.17, respectively), while the sensitivity of SE-BOLD is approximately three times lower than that of GE-BOLD at 9.4 T [26]. Further, the spatial specificity of SE-BOLD fMRI is still lower than that of other fMRI contrasts: 0.4–1.0 for the early negative GE-BOLD [26,30], 0.76 for CBF with ASL-perfusion fMRI [29] and 0.48 for CE-CBV fMRI [25]. The sensitivities of the BOLD initial dip and ASL-perfusion fMRI signals are very low compared with positive GE-BOLD, while the sensitivity of CE-CBV fMRI is 1.6 times higher than that of GE-BOLD at 9.4 T [31]. Thus, CE-CBV fMRI is the method of choice for high-resolution fMRI, if contrast agents can be used.

Successful mapping of iso-orientation columns using fMRI suggested that the blood supply can be regulated within approximately less than 0.5 mm. However, it was unknown whether this neural specificity originated from penetrating arteries [32], and/or microvessels, like small arterioles and capillaries. This issue can be further investigated in the cortical layer model since penetrating arteries that span across multiple layers cannot resolve layer-specific changes, unlike actively regulated microvessels.

## 5. Spatial specificity of fMRI examined in the cortical layer model

The neocortex consists of six layers that vary in thickness depending on the brain region and species. For example, the thickness of the cat primary visual cortex is approximately 1.7 mm with each layer having a thickness of approximately 100–300 µm. If the blood supply to each layer is discretely regulated by microvessels and fMRI can resolve submillimetre-scale domains, then fMRI may capture neuronal processing in each layer. Maximal neuronal activity is expected to occur in layer IV (approx. 300 µm thickness) of the cat primary visual cortex during visual stimulation.

**Figure 3.**
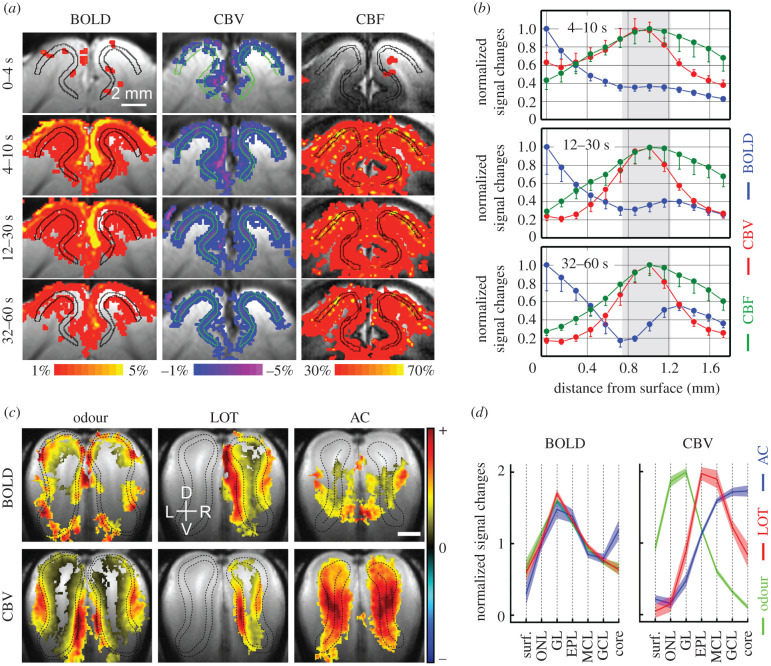
Time-dependent laminar-resolution fMRI of animal models. (*a*) Time-dependent laminar-resolution BOLD (left columns), CE-CBV (middle) and CBF fMRI (right) maps of the cat visual cortex from four different time periods after the 60 s visual stimulation onset. Statistically active voxels were overlaid on the coronal anatomical slice. The black and green contours indicate the middle cortical layer. Decreased CE-CBV signals correspond to increased CBV (vasodilation). Note the lack of pial vessel activation in CBV and CBF images. CBF studies were performed separately from BOLD and CBV fMRI and had a twofold decrease in spatial resolution. Provided courtesy of Dr Tao Jin. (*b*) Time-dependent changes of the laminar response profiles for BOLD, CBV and CBF fMRI signals obtained from the cat primary visual cortex. For comparison across modalities, normalized cortical profiles of BOLD (blue traces), CBV (red) and CBF (green) were plotted for three time periods (top left corner). Grey bars: middle layer, presumably layer IV; error bars: s.e.m. (*n* = 4 cats). Adapted from [33]. (*c,d*) BOLD and CE-CBV fMRI maps (*c*) and laminar profiles (*d*) of the rat olfactory bulb evoked by discretely stimulating three different input layers (GL, EPL or GCL by odour stimulation, right lateral olfactory tract (LOT) microstimulation or anterior commissure (AC) microstimulation, respectively). (*c*) For both odour and AC stimulations, both olfactory bulbs were activated, while only the right bulb was activated during right LOT stimulation. Black dashed contour boundaries of the synaptically evoked layer: GL for odour, EPL for LOT and GCL for AC stimulations; scale bar: 1 mm; D: dorsal; V: ventral; R: right; L: left. (*d*) While peak BOLD responses were located at the same layer regardless of which input layer was stimulated, the peak CBV responses matched the layers of preferentially evoked synaptic activity independent of the baseline blood volume condition. Abbreviations: surf., bulb surface; ONL, olfactory nerve layer; GL, glomerular layer; EPL, external plexiform layer; MCL, mitral cell layer; GCL, granule cell layer. Adapted from [34].

To examine whether the largest fMRI responses were also evoked in layer IV, the spatio-temporal dynamics of GE-BOLD, CE-CBV and ASL-perfusion fMRI signals evoked by a 60 s full-field square-wave grating stimulation were compared [33] (figure 3). Initially, a small positive BOLD response appeared in layer IV (see 0–4 s), but later spread across the layers until the largest responses were at the cortical surface (figure 3*a,b*). This fast, layer IV-specific BOLD response was more convincingly demonstrated by line scanning BOLD fMRI in the rat primary somatosensory cortex [35]. The poor layer specificity of the later GE-BOLD fMRI responses was partly due to the spreading of non-specific pial vein contributions (see yellow voxels at the cortical surface in figure 3*a*). Although large vessel contributions can be significantly reduced by SE-BOLD fMRI, the response profile still did not show peak responses in layer IV [36].

On the contrary, CE-CBV fMRI responses first appeared in, presumably, the penetrating arterioles (see 0–4 s), but then became increasingly more specific to layer IV over time (figure 3*a,b*), which is consistent to our previous observations with orientation-specific CE-CBV fMRI (figure 2*b*). Thus, different temporal dynamics of penetrating arteriole and microvascular responses could be responsible for this increasing specificity. The later layer-specific CE-CBV fMRI responses were similarly detected in both GE and SE techniques [36], indicating a microvascular origin. Evoked CBV responses can also be measured non-invasively with vascular space occupancy (VASO) fMRI. VASO responses induced by visual stimulation peaked in layer IV, which is consistent with CE-CBV fMRI [37].

Since CBV and CBF responses are coupled in the area where increased neuronal activity is observed, evoked CBF responses were also expected to be layer-specific [38]. Early CBF responses in the cat visual cortex were not observed during 0–4 s, likely owing to its poor sensitivity. However, unlike CBV, CBF localizations to layer IV did not change over time, likely because the ASL fMRI signal mostly originates from the perfused tissue surrounding microvessels.

The CBV and CBF fMRI results suggest that the blood supply is discretely regulated in neuronally active layers. However, this ‘apparent’ layer specificity can be due to the underlying evoked neuronal activity or the baseline microvascular blood volume since layer IV has both the highest neural activity and the highest baseline CBV. To investigate whether the highest haemodynamic responses genuinely reflect the highest neural activity, we adopted the rat olfactory bulb model to selectively and independently evoke synaptic activities in three distinct layers with three different stimulations, respectively [39]. The baseline microvascular volume here is highest in the superficial input layer and declines with increasing depth [14]. While maximal BOLD responses always appeared in the same superficial layer regardless of which layer was stimulated, the maximal CE-CBV responses consistently peaked in the elicited layer independent of the baseline blood volume (figure 3*c,d*). Further, the CBV response to a single-layer stimulation spread by about 100 µm [40]. This result definitely clarifies that the blood supply to each layer is discretely regulated by microvessels responding to neuronal activities and, therefore, cortical layer-specific processing can be resolved by CBV and CBF fMRI.

## 6. Insights into neurovascular coupling and outstanding questions

**Figure 4.**
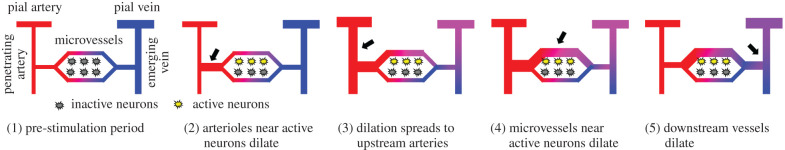
Schematics of expected dynamic vascular responses induced by neuronal activities. (1) Baseline. (2) Sensory stimulation increases CBF and CBV near the neuronally active site by dilation of pre-capillary arterioles and/or capillaries (arrow). (3) Then, local arterial vasodilation backpropagates toward the pial arteries (arrow), while oxygenated blood draining increases in the emerging and pial veins. (4) Later, microvessels near the active neurons dilate further (arrow), improving the spatial localization to them. (5) During prolonged stimulation, small vasodilations occur in largely distributed draining venules (arrow), while vasodilation in the non-specific feeding arteries decreases. This homeostatic pressure control preserves vasodilation in microvessels and, consequently, further improves the detection of the neural-specific CBV signal changes.

To interpret time-dependent fMRI with compartment-specific vascular responses, it is important to consider that BOLD, CBV and CBF fMRI mainly stem from venous vessels (including capillaries), arterial vessels (including capillaries) and parenchyma near capillaries, respectively. Note that CBV and CBF can also be determined by an isosbestic OISI wavelength and laser Doppler flowmetry, respectively, at mesoscales, and by vessel diameter and red blood cell (or plasma) flux in two-photon microscopy, respectively, at microscales. Interpretation of column- and layer-specific fMRI data is summarized in figure 4. Initially, sensory stimulation increases CBF and CBV near the neuronally active site by dilation of pre-capillary arterioles and/or capillaries [41]. Next, the local arterial vasodilation backpropagates toward the pial artery [42], and draining spreads to the emerging and pial veins. Later, microvessels near the neurons dilate further, while responses in parent arteries decrease. To further link mesoscopic fMRI and microscopic studies, unresolved outstanding issues are listed below:

— BOLD responses evoked by sensory stimulation first appeared in layer IV [33,35], and initial CBF and CBV changes were expected to be the same [43]. Two-photon measurements, however, showed the initial vasodilation in response to electrical forepaw stimulation in layer VI [42]. This discrepancy still needs to be evaluated, but it may be explained by a difference in the stimulation context or modality that changed the discrete input timing to layers IV, V and VI from different thalamic nuclei.
— Vasodilation can be controlled by pericytes, pre-capillary sphincters, smooth muscle cells and/or endothelial cells in the neurovascular unit. For example, pericytes are spatially isolated contractile cells on capillaries [44], but whether they actively regulate blood flow is debatable [45,46]. Alternatively, *N*-methyl-d-aspartate (NMDA) receptors on endothelial cells may play a role in active capillary dilation [47]. In our CE-CBV fMRI studies, the early vasodilation was likely in penetrating arteries. To determine the laminar origins of CBV responses, further dynamic CBV fMRI investigations with higher spatial and temporal resolutions are required.
— Later CBV responses appear to be more specific to the neuronally active sites [25,48], which could be due to ‘passive’ dilation of capillaries and/or ‘active’ control of small arterioles and capillaries. The exact source of this increased localization at later time points during longer stimulations is unknown. One possibility is active vascular control by astrocytes, based on greater astrocytic calcium increases that were slower than the vascular onset time [49]. Another hypothetical explanation is the passive control of ‘specific’ microvessels [25]. When arterial vessels dilate, the upstream segmental blood pressure difference between arteries and microvessels decreases, while downstream segmental blood pressure difference between microvessels and veins increases, further inducing dilations of small arterioles, possibly capillaries, and later in venous vessels. Dilation of these downstream vessels decreases the total vascular resistance, thus, a coordinated reduction in upstream arterial dilation maintains a constant CBF and arterial–venous blood pressure differences at the localized capillaries. This will further improve CBV fMRI localization. Further neurovascular optical studies with longer stimulations are necessary to better interpret the fMRI data.
— Time-dependent fMRI responses among different layers or regions may reflect the sequence of neuronal activities if the measured fMRI responses are highly sensitive to capillaries. Sensory stimulation first evokes neuronal activity in granular layer IV and subsequently in the supra- and infragranular layers. However, the time difference of these fMRI responses between layers (hundreds of milliseconds) [35] is much longer than that of the neuronal responses (milliseconds) [50]. This non-linear temporal relationship may be due to underlying haemodynamic regulation mechanisms. For example, the response latency of layer IV neurons to individual pulses was constant (approx. 10 ms) regardless of the time from train onset, while response latencies of neurons in supra- and infragranular layers cumulatively increased with time (approx. 15–40 ms during the 15 s long stimulation). Therefore, if cumulatively synchronized (coherent) neuronal input is necessary to induce haemodynamic responses at the mesoscale, this may explain the delayed fMRI responses in supra- and infragranular layers. Systematic studies are necessary to understand the temporal haemodynamics relative to the population of neural activities in different layers or regions.
— The haemodynamic peak response should shift to different layers in a time-dependent manner owing to recurrent laminar connections. However, for unknown reasons, only the first synaptically activated layer induces the highest CBV response [39]. The haemodynamic response may be related to the magnitude of synchronized neural population activities, and the first synaptically activated layer may have the largest synchronized activity, as mentioned above [50]. Also, a subtype of inhibitory cells may be involved in sharpening the localization to the first synaptic site [51]. Further studies are warranted.

## 7. Implications for human high-resolution fMRI

Systematic animal research is used to understand the neural specificity of evoked vascular responses and will provide critical information for human fMRI design and interpretation. However, because of physiological differences between humans and animals, caution should be exercised with direct human translation. First, anaesthesia is often used for fMRI with animal models, which can modulate vascular tone, baseline neuronal activity, neuromodulatory inputs, astrocytic activity, etc. As a result, the magnitude and dynamics of the fMRI signals will be different. However, if the localized neuronal activity is preserved under anaesthesia, which is often true in primary sensory cortices, the spatial localization of each fMRI contrast should also be preserved. Second, the density of penetrating arteries varies depending on the species. Assuming each penetrating arteriole is controlled independently, a higher density of penetrating arterioles in humans will increase functional localization at higher spatial resolutions using arterial vessel-based CBV and CBF techniques. By contrast, a higher density of emerging veins may not improve the localization of BOLD responses because the emerging veins collect drainage from remote areas. Third, CBF responses can be evoked by synaptic and/or spiking activity. Since the costs of spiking and synaptic activities seem different among species [52,53], neurometabolic and neurovascular coupling may also be different among species. However, with general findings, such as localization, neurovascular coupling mechanisms and the biophysics of BOLD fMRI should be preserved.

For non-invasive high-resolution fMRI studies of the human brain (see [54] for review), conventional GE-BOLD fMRI is most widely used for its high sensitivity. As is well known, non-specific draining vessels contribute significantly to BOLD fMRI even at high magnetic fields and mask specific changes in microvessels. Therefore, such BOLD studies require differential methods to obtain neuronal-specific signals under the assumption that the common responses, such as from pial veins, are removed by simple subtraction or saturated by continuous cyclic stimulation. However, this approach cannot be used for many cases. Therefore, other techniques that have superior spatial specificity should be considered. Based on our studies described above, non-invasive CBF or CBV techniques can be a better choice. High-field ASL fMRI for blood flow measurements is advantageous for its long *T*_1_ of blood, but it is difficult to implement the most advanced techniques owing to their large specific absorption rates. Consequently, the sensitivity of CBF fMRI may be insufficient for high-resolution fMRI. Endogenous CBV-weighted VASO fMRI is a promising alternative, although its coverage is limited. Indeed, combined with a sophisticated stimulation paradigm, modulations of layer-specific CBV responses have been beautifully demonstrated in humans using VASO [55]. Regardless of the technique used, it is critical to enhance the microvascular sensitivity with ultra-high magnetic fields, highly sensitive array coils and advanced imaging techniques.

## 8. Conclusion

As technology improves and submillimetre human fMRI becomes practical, animal models become increasingly important to validate its limits on neural specificity. Based on our functional columnar mapping and layer-specific studies, we recommend exogenous CE-CBV fMRI for studying animal brain functions as a promising alternative to conventional BOLD fMRI owing to its superior sensitivity, high spatial specificity, and straightforward physiological signal source. Since fMRI can be easily combined with many other techniques, such as optogenetics, electrophysiology, optical imaging, pharmacology and histology, we believe that high-resolution fMRI with animal models continues to provide valuable insights into neurovascular coupling and, thus, improves the interpretability of fMRI maps.

## Data accessibility

This article has no additional data.
